# Efficacy of virtual reality-based cognitive behavioral group therapy in enhancing emotional well-being and quality of life in Parkinson’s disease: A randomized controlled trial

**DOI:** 10.1016/j.prdoa.2025.100316

**Published:** 2025-03-11

**Authors:** Gooya Tayyebi, Farnaz Asadiof, Bahar Hashempour, Mohsen Lotfi, Mostafa Taheri, Mahdi Naeim

**Affiliations:** a Department of Psychiatry, Member of the Faculty of Mazandaran University of Medical Sciences, Mazandaran, Iran; b Educational Psychology Department, Payame Noor University, Tehran, Iran; c Department of Psychology, Islamic Azad University, Naein Branch, Isfahan, Iran; d Department of Clinical Psychology, University of Social Welfare and Rehabilitation Sciences, Tehran, Iran; e Department of Critical Care and Emergency Nursing, Zanjan Nursing and Midwifery School, Zanjan University of Medical Sciences, Zanjan, Iran; f Department of Research, Psychology and Counseling Organization, Tehran, Iran

**Keywords:** Virtual reality, Cognitive behavioral therapy, Parkinson’s disease, Emotional well-being, Quality of life, HADS, PDQ-39

## Abstract

**Objective:**

This study evaluates the effectiveness of virtual reality-based cognitive behavioral group therapy (VR-CBGT) in improving emotional well-being and quality of life in individuals with Parkinson’s disease.

**Methods:**

A randomized controlled trial was conducted in 2023 at Roozbeh Hospital, Tehran, with 90 Parkinson’s patients. Participants were randomly assigned to an intervention group receiving 12 VR-CBGT sessions over three months with a control group receiving standard medical care without psychological intervention. Emotional well-being was assessed using the Hospital Anxiety and Depression Scale (HADS), and quality of life was measured with the Parkinson’s Disease Questionnaire-39 (PDQ-39) before and after the intervention. Data were analyzed using Multivariate Analysis of Covariance (MANCOVA) in SPSS-25.

**Results:**

The intervention group demonstrated a significant improvement in emotional well-being (HADS-total score reduction of 7.2 points, P < 0.001) and quality of life (PDQ-39 total score improvement of 12.5 %, P = 0.002) compared to the control group. VR-CBGT explained 18 % of the variance in emotional well-being and 26 % in quality of life.

**Conclusion:**

These findings highlight VR-CBGT as an effective complementary intervention for enhancing psychological health and overall quality of life in Parkinson’s patients. The immersive nature of VR fosters engagement and facilitates cognitive and emotional processing, supporting its integration into multidisciplinary treatment approaches.

## Highlights

• VR-CBGT enhances emotional well-being and quality of life in Parkinson’s patients.
• This method explains 22% of emotional well-being changes and 38% of quality of life improvements.
• Virtual reality boosts therapeutic engagement and cognitive-emotional processing.
• Findings support VR integration into psychological therapies and multidisciplinary care.

## 1 Introduction

Parkinson’s disease (PD) is a chronic and progressive neurodegenerative disorder that ranks as the second most prevalent neurological condition after Alzheimer’s disease in aging populations. It primarily affects the central nervous system and is one of the leading causes of disability, particularly among individuals aged 65 and older, with a prevalence rate of approximately 1.8 % in this age group [1,2]. PD is characterized by the degeneration of dopaminergic neurons in the brain, leading to a range of motor and non-motor symptoms [3]. While motor symptoms such as resting tremors, bradykinesia, rigidity, and postural instability are the hallmark of PD, patients also experience significant cognitive, emotional, and social challenges [4].

The comprehensive impact of PD on physical, emotional, and social functioning underscores the need for integrated care strategies that address not only the motor symptoms but also the psychological and social dimensions of the disease. In particular, the emotional well-being of individuals with PD is frequently compromised, with many patients reporting diminished quality of life (QoL) due to both psychological and physical impairments [5,6]. QoL is a multidimensional construct encompassing physical health, psychological well-being, social relationships, and functional ability. In PD patients, the presence of motor deficits, cognitive challenges, and emotional disturbances leads to significant reductions in these domains, negatively impacting their overall QoL [7,8].

Among psychological interventions, cognitive behavioral therapy (CBT) has emerged as an evidence-based approach for improving emotional well-being and enhancing QoL in various patient populations. CBT is a structured therapeutic approach that targets maladaptive thoughts and behaviors, with the goal of improving emotional regulation and psychological functioning [9]. Group-based CBT, in particular, offers a cost-effective framework that fosters a supportive therapeutic environment, which may be particularly beneficial for individuals managing chronic conditions such as Parkinson’s disease [10]. Recent studies have supported the utility of CBT in addressing the emotional and cognitive difficulties associated with PD, demonstrating improvements in psychological health and overall QoL [11,12].

Despite the established effectiveness of CBT, recent advances in technology have led to the integration of virtual reality (VR) with traditional therapeutic approaches, offering promising new avenues for intervention. VR provides immersive, interactive environments that enhance patient engagement and facilitate experiential learning, allowing for more effective cognitive and emotional processing in a controlled setting [13]. When combined with CBT, VR can create realistic scenarios that promote behavioral and cognitive practice, potentially amplifying the therapeutic benefits of CBT. This combination has shown promise in treating various psychological issues, including depression, anxiety, and stress, in diverse clinical populations [14]. However, its application in Parkinson’s disease, particularly in a group therapy setting, remains underexplored.

This study seeks to address this gap by evaluating the efficacy of virtual reality-based cognitive behavioral group therapy (VR-CBGT) in improving emotional well-being and quality of life in individuals with Parkinson’s disease. By merging the traditional framework of CBT with cutting-edge VR technology, this research aims to enhance psychological interventions and improve the overall well-being of PD patients. Through this innovative approach, the study seeks to contribute to the development of more effective, engaging, and accessible treatment options for enhancing both the psychological health and quality of life in individuals with Parkinson’s disease.

## 2 Method

**Fig. 1:**
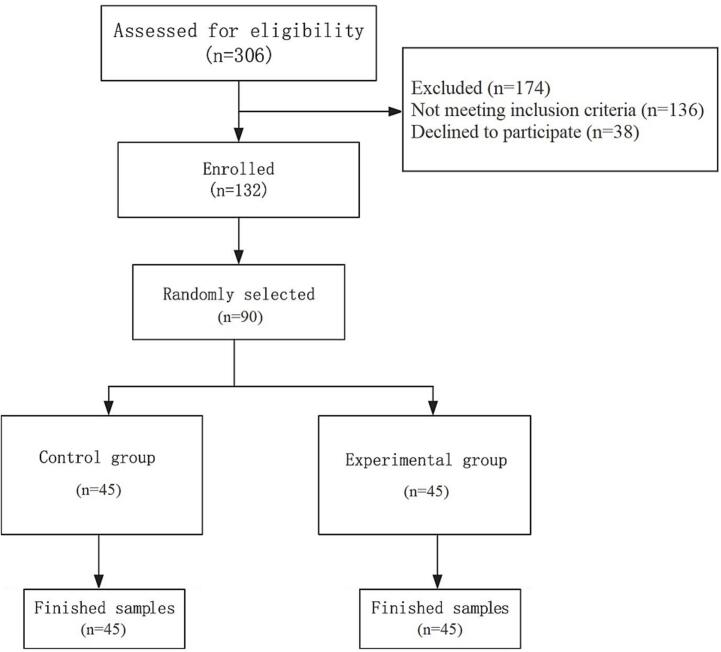
Study CONSORT diagram.

This study employed a randomized controlled trial (RCT) with a pre-test and post-test design, incorporating a control group. The statistical population included individuals diagnosed with Parkinson's disease who were referred to Roozbeh Hospital, Tehran, in 2023. After obtaining informed consent, participants were recruited through a convenience sampling method. A total of 90 Parkinson’s patients who met the inclusion criteria were selected and randomly assigned to either the experimental group (45 participants) or the control group (45 participants). The study follows the CONSORT guidelines for reporting randomized trials. A flow diagram illustrating participant enrollment and group allocation is provided in Fig. 1.

## 3 Inclusion and exclusion criteria

*Inclusion Criteria:*

• Diagnosis of Parkinson’s disease confirmed by a neurologist and psychiatrist.
• Written consent to participate in the study.
• No acute or chronic physical illnesses, as per medical records.
• No use of psychiatric medications, as verified by medical records.
• At least a middle school education level.
• Currently receiving prescribed medication for Parkinson’s disease.
• Mild to moderate emotional distress, including anxiety or depressive symptoms, verified through clinical evaluations.

*Exclusion Criteria:*

• Missing more than two therapy sessions.
• Non-compliance with therapeutic tasks or instructions.
• Withdrawal of consent at any stage of the study.

## 4 Session Content

### 4.1 Session 1: Introduction to the therapy framework

• Overview of the group dynamics, therapy rules, and the integration of VR with Cognitive Behavioral Therapy (CBT).
• Discussion of Parkinson’s disease, focusing on the non-motor symptoms (especially anxiety and depression), and the impact on emotional well-being and quality of life.
• Explanation of how VR will be used to facilitate engagement and support cognitive and emotional processing during therapy.

### 4.2 Session 2: Relaxation techniques and mindfulness with VR

• Introduction to relaxation techniques (e.g., deep breathing, progressive muscle relaxation) and mindfulness exercises aimed at reducing stress and improving emotional regulation.
• Participants practice these techniques in calming, immersive VR environments designed to reduce physiological anxiety responses and promote emotional well-being.

### 4.3 Session 3: Cognitive distortions and challenging negative thoughts

• Identification of automatic negative thoughts (ANTs) linked to anxiety and depression, with a focus on how these distortions affect emotional resilience.
• VR simulations help participants visualize these thoughts in real-life scenarios, providing a safe space to challenge and replace cognitive distortions, promoting a healthier emotional outlook.

### 4.4 Session 4: Assertiveness training and anger management

• Focus on assertiveness and anger management in the context of Parkinson’s disease-related stressors and emotional regulation.
• VR role-playing scenarios allow participants to practice interpersonal skills and manage emotional reactivity in social situations, contributing to improved social interactions and overall emotional well-being.

### 4.5 Session 5: Cognitive restructuring

• Introduction to cognitive restructuring techniques, teaching participants to replace negative or maladaptive thoughts with more adaptive thinking patterns.
• Participants engage in VR environments simulating challenging social or daily situations, helping them practice cognitive restructuring in a safe and supportive space.

### 4.6 Sessions 6**–**7: Exposure to Anxiety-Inducing situations

• Gradual exposure to anxiety-provoking VR scenarios (e.g., crowded places, unfamiliar environments) to reduce avoidance behaviors and increase emotional resilience.
• Integration of cognitive rehabilitation techniques to enhance emotional regulation, fostering improved coping with external stressors and promoting long-term emotional well-being.

### 4.7 Sessions 8**–**9: Problem-solving and coping skills

• Introduction to problem-solving strategies and coping mechanisms, with a focus on practical applications for daily life.
• VR-based tasks are used to help participants navigate challenges, while simultaneously working on enhancing self-efficacy and building a positive self-narrative about their abilities and life goals.

### 4.8 Session 10: Review and integration of skills

• A session dedicated to reviewing and integrating the strategies learned, with participants reflecting on their emotional progress and sharing insights within the group.
• VR settings provide a space for practice and reinforcement, with participants discussing the real-life application of the learned skills in managing anxiety and depression.

### 4.9 Sessions 11**–**12: Consolidation and long-term support strategies

• Consolidation of the therapeutic gains made during the sessions, focusing on long-term emotional and social support strategies.
• Participants practice these strategies in VR environments and receive individualized feedback on their progress, promoting ongoing improvement in emotional well-being and quality of life even after therapy concludes.

### 4.10 Control group

• The control group continued their standard medical treatment for Parkinson’s disease without psychological intervention during the study period. After completing the study, participants in the control group were placed on a waitlist to receive the VR-CBGT intervention.

### 4.11 Tools

#### 4.11.1 Hospital anxiety and depression Scale (HADS)

The HADS is a widely used 14-item tool designed to assess both anxiety and depression symptoms. Scores range from 0 to 42, with higher scores indicating more severe anxiety or depression. The HADS is specifically validated for use in medical populations and has shown good reliability (Cronbach’s alpha = 0.85 for both anxiety and depression subscales) [15]. This tool was selected to measure emotional well-being due to its established effectiveness in clinical settings, particularly among individuals with chronic health conditions such as Parkinson's disease.

#### 4.11.2 Parkinson’s disease Questionnaire-39 (PDQ-39)

The PDQ-39 is a 39-item questionnaire designed to assess quality of life specifically in individuals with Parkinson’s disease. It covers 8 dimensions of health, including mobility, activities of daily living, emotional well-being, stigma, social support, and communication. Higher scores reflect a lower quality of life. The PDQ-39 is considered the gold standard for assessing quality of life in Parkinson's disease and has demonstrated excellent internal consistency (Cronbach’s alpha = 0.92) [16].

### 4.12 Procedure

Participants in both the experimental and control groups completed the HADS and PDQ-39 at two points: before the intervention (pre-test) and after the intervention (post-test). The experimental group received 12 sessions of VR-CBGT over three months at Roozbeh Hospital, while the control group continued their usual Parkinson’s disease care without any psychological intervention. Sessions were facilitated by licensed therapists and supervised by clinical psychologists, ensuring that all therapeutic techniques were delivered consistently and effectively.

The VR-CBT intervention focused on improving emotional well-being (e.g., through relaxation, cognitive restructuring, and problem-solving strategies) and enhancing quality of life (e.g., through social engagement and coping strategies). The experimental group used immersive VR environments to practice skills in a controlled and interactive setting, designed to help improve psychological functioning and foster emotional resilience.

### 4.13 Data analysis

Data were analyzed using SPSS-25 software. Descriptive statistics, such as mean and standard deviation, were used to summarize the data. To test the effectiveness of VR-CBGT on improving emotional well-being and quality of life, Multivariate Analysis of Covariance (MANCOVA) was conducted. This method allowed for controlling baseline differences in both anxiety and depression, as well as ensuring that any changes in the outcomes were attributed to the intervention rather than external factors.

## 5 Results

This study included 90 individuals diagnosed with Parkinson’s disease, focusing on emotional well-being and quality of life. Participants were randomly assigned to either the experimental group (45 participants), which received Virtual Reality-Based Cognitive Behavioral Group Therapy (VR-CBT), or the control group (45 participants), which received standard medical care. Prior to conducting the main analyses, assumptions necessary for Multivariate Covariance Analysis (MANCOVA) were evaluated:

The Kolmogorov-Smirnov test revealed no significant deviation from normality for any variable (P > 0.05), confirming the assumption of normal distribution.

Both the M−Box test and Levene’s test showed no significant differences (P > 0.05), supporting the assumptions of equality of covariance matrices and homogeneity of variances, respectively.

### 5.1 Participant demographics

The demographic characteristics of participants in both the experimental and control groups were similar, ensuring the comparability of groups. The distribution of gender and education level is provided in Table 1.

**Table 1:** Frequency and percentage distribution of gender and education.

Group	Male (N)	Female (N)	Middle School Diploma (N)	Diploma (N)	Post-graduate Diploma (N)	Bachelor’s Degree (N)
Experimental	Male: 24 (53 %)	Female: 21 (47 %)	20	21	2	2
Control	Male: 24 (53 %)	Female: 21 (47 %)	21	20	2	2

According to Table 1, the distribution of gender and educational levels in the experimental and control groups was similar, ensuring group comparability.

### 5.2 Descriptive statistics

Table 2 presents the mean and standard deviation of pre-test and post-test scores for emotional well-being (HADS) and quality of life (PDQ-39) in both groups.

**Table 2:** Mean and standard deviation of pre-test and post-test scores for emotional well-being and quality of life.

Group	N	Emotional Well-being (HADS)	Quality of Life (PDQ-39)
		Pre-test (M ± SD)	Post-test (M ± SD)
Experimental	45	28.15 ± 5.20	15.35 ± 4.10
Control	45	27.90 ± 5.10	26.30 ± 5.60

According to Table 2, the experimental group demonstrated a significant reduction in both emotional well-being and quality of life scores. The post-test emotional well-being score in the experimental group (M = 15.35, SD = 4.10) was significantly lower than the control group (M = 26.30, SD = 5.60). Similarly, quality of life (PDQ-39) showed significant improvement in the experimental group, with a mean score reduction from 71.80 to 61.20, compared to a smaller change in the control group (from 72.50 to 70.90).

### 5.3 Multivariate test results

The results of the Multivariate Analysis of Covariance (MANCOVA) for the combined effect of the therapy method on emotional well-being and quality of life are shown in Table 3.

**Table 3:** Results of multivariate test for emotional well-being and quality of life.

Independent Variable	Test	Wilks’ Lambda	F	Sig.	Eta^2^
Therapy Method	Pillai’s Trace	0.435	21.60	0.0005	0.75
	Wilk’s Lambda	0.543	21.60	0.0005	0.73
	Hotelling’s Trace	1.035	21.60	0.0005	0.74
	Roy’s Largest Root	1.180	21.60	0.0005	0.76

The results from the multivariate tests confirmed that the group therapy method (VR-CBT) had a significant effect on both emotional well-being and quality of life (P ≤ 0.0005). The Eta^2^ values (ranging from 0.73 to 0.76) indicate a large effect size, demonstrating that therapy accounted for a substantial portion of the variance in the improvements observed in both emotional well-being and quality of life.

### 5.4 Covariance analysis results for emotional well-being

Table 4 shows the results of the covariance analysis for the impact of VR-CBT on emotional well-being (HADS) scores.

**Table 4:** Results of covariance analysis of VR-CBT and control groups on emotional well-being (HADS)**.**

Source	Sum of Squares	df	Mean Square	F	Sig.	Eta^2^
Pre-test	162.830	1	162.830	42.360	0.0005	0.61
Group	42.770	1	42.770	10.560	0.002	0.22
Error Variance	126.570	27	4.685			

As shown in Table 4, VR-CBT significantly improved emotional well-being, with a reduction in HADS scores (P = 0.002, F = 10.560). The Eta2 value of 0.22 indicates that 22 % of the variance in emotional well-being changes can be attributed to the intervention, supporting its effectiveness in enhancing emotional health in Parkinson's patients.

### 5.5 Covariance analysis results for quality of life

Table 5 presents the covariance analysis results for the impact of VR-CBT on quality of life (PDQ-39) scores.

**Table 5:** Results of covariance analysis of VR-CBT and control groups on quality of life (PDQ-39)**.**

Source	Sum of Squares	df	Mean Square	F	Sig.	Eta^2^
Pre-test	31285.550	1	31285.550	298.790	0.0005	0.83
Group	2451.760	1	2451.760	22.030	0.0001	0.38
Error Variance	271.560	27	10.060			

Table 5 demonstrates that VR-CBT significantly improved quality of life, with a reduction in PDQ-39 scores (P = 0.0001, F = 22.030). The Eta2 value of 0.38 indicates that 38 % of the variance in quality of life changes can be attributed to the intervention, highlighting its effectiveness in improving the quality of life of patients with Parkinson's disease.

## 6 Discussion

Patients with Parkinson’s disease (PD) frequently face significant psychological challenges, including anxiety, depression, and diminished emotional well-being, which negatively impact their quality of life. These symptoms are often exacerbated by the chronic nature of the disease and the associated stressors, highlighting the need for effective therapeutic interventions. This study investigated the impact of Virtual Reality-Based Cognitive-Behavioral Group Therapy (VR-CBGT) on improving emotional well-being and quality of life in individuals with Parkinson’s disease.

The results of this study demonstrated that VR-CBGT significantly enhanced emotional well-being and quality of life in the experimental group compared to the control group. Specifically, the intervention led to substantial improvements in emotional regulation, reduced depressive and anxious symptoms, and increased overall satisfaction with life. These findings are consistent with previous research supporting the efficacy of cognitive-behavioral therapy (CBT) in addressing mental health issues in chronic conditions, including Parkinson’s disease [4,11,12,17,18]. For example, Dobkin et al. (2020) showed that CBT improved emotional well-being by reducing depression and anxiety while enhancing quality of life in Parkinson’s patients [11]. Similarly, Berardelli et al. (2018) observed that CBT alleviated depression, anxiety, and non-motor symptoms in PD patients, leading to an overall improvement in their quality of life [12]. Piers et al. (2023) also found that CBT had lasting effects on mental health, improving mood, cognitive functioning, and social interaction [4].

The positive impact of VR-CBGT in this study can be attributed to its multidimensional approach. The integration of virtual reality (VR) into CBT creates an immersive, engaging environment that enhances therapeutic participation. VR enables patients to experience real-life scenarios in a controlled and supportive setting, which facilitates skill acquisition and emotional processing. This immersive experience allows participants to practice and internalize coping strategies, bridging the gap between therapy and real-life challenges [[15], [16], [17]].

Furthermore, the cognitive-behavioral framework targets maladaptive thought patterns and behaviors that contribute to emotional distress. In group therapy settings, individuals benefit from shared experiences and collective problem-solving, fostering a sense of community and reducing isolation. The group dynamic in CBT promotes a supportive environment where participants can learn from one another and gain motivation to improve their mental health [[18], [19], [20], [21], [22]].

The structured nature of CBT, coupled with the use of VR, equips participants with practical skills, including:

Relaxation techniques and stress management strategies, such as mindfulness exercises, to enhance emotional resilience [23].

Cognitive restructuring techniques to challenge negative thoughts and adopt more adaptive thinking patterns [24,25].

Goal-setting and planning skills aimed at improving daily functioning and overall quality of life [26,27].

Problem-solving strategies that boost confidence in managing social and personal challenges [28].

The VR component amplifies these therapeutic strategies by providing a dynamic and interactive medium where participants can practice coping skills in a safe and engaging environment. This combination of CBT and VR aligns with current evidence-based practices, emphasizing both technological innovation and traditional therapeutic principles.

In conclusion, this study highlights the effectiveness of VR-CBGT as an innovative approach to improving emotional well-being and quality of life for individuals with Parkinson’s disease. The integration of VR not only enhances therapeutic engagement but also provides patients with valuable tools to manage their condition, making the therapy both practical and impactful. These findings suggest that VR-CBGT could be a promising addition to the treatment strategies for Parkinson’s disease, offering significant benefits for the psychological and social well-being of patients.

## 7 Conclusion

This research underscores the potential of virtual reality-based cognitive-behavioral group therapy (VR-CBGT) as an effective intervention for improving emotional well-being and quality of life in patients with Parkinson's disease. The dual benefits of this approach—enhancing psychological health and fostering improvements in daily functioning—highlight its value for healthcare providers, including therapists and clinical psychologists, in addressing the complex psychological challenges faced by these patients. The immersive nature of VR combined with cognitive-behavioral techniques offers a unique and engaging method for managing mental health, making it a promising tool for long-term therapeutic interventions.

## 8 Limitations and recommendations

Despite the encouraging findings, there are several limitations to this study that should be addressed in future research:

Absence of a Follow-Up Phase: The lack of a follow-up period in this study limits our understanding of the long-term sustainability of the intervention’s effects on emotional well-being and quality of life. Future studies should include follow-up assessments to evaluate whether the benefits of VR-CBGT are maintained over time and to determine the lasting impact on patients' mental health.

Self-Reported Data: The reliance on self-reported questionnaires for measuring depression, anxiety, and quality of life may introduce response bias, as participants may not always provide accurate assessments of their psychological and emotional states. Future research could incorporate additional objective measures, such as clinician ratings or biomarkers, to complement self-reported data.

Restricted Sample Population: This study was conducted in a single center, which limits the generalizability of the findings to a broader population. Including a more diverse sample across multiple centers would enhance the external validity of the results and provide insights into the effectiveness of VR-CBGT across different patient demographics.

## 9 Suggestions for future research

Comparative Efficacy of VR-CBGT: Future studies should explore the comparative efficacy of VR-CBGT against other therapeutic modalities, such as schema therapy or mindfulness-based cognitive therapy, to determine the relative benefits of these approaches in improving emotional well-being and quality of life in patients with chronic conditions.

Broader Application in Other Chronic Diseases: The potential of VR-CBGT should be evaluated in other chronic conditions such as cancer, diabetes, or cardiovascular diseases, to examine its applicability in improving emotional well-being and quality of life for patients with different health challenges.

Cost-Effectiveness and Feasibility: Investigating the cost-effectiveness and feasibility of implementing VR-CBGT in various clinical settings, including community-based healthcare centers and rehabilitation facilities, would provide valuable insights into the practicalities of incorporating this therapeutic approach into routine clinical practice.

## CRediT authorship contribution statement

**Gooya Tayyebi:** Conceptualization, Data curation, Formal analysis, Funding acquisition, Investigation, Methodology, Project administration, Resources, Software, Supervision, Validation, Visualization, Writing – original draft, Writing – review & editing. **Farnaz Asadiof:** Conceptualization, Formal analysis, Investigation, Project administration, Software, Validation, Writing – original draft, Writing – review & editing. **Bahar Hashempour:** Data curation, Funding acquisition, Methodology, Resources, Software, Validation, Writing – original draft. **Mohsen Lotfi:** Data curation, Formal analysis, Methodology, Resources, Software, Validation, Visualization. **Mostafa Taheri:** Conceptualization, Data curation, Formal analysis, Funding acquisition, Investigation, Methodology, Project administration, Resources, Software, Supervision, Validation, Visualization, Writing – original draft, Writing – review & editing. **Mahdi Naeim:** Writing – review & editing, Writing – original draft, Visualization, Validation, Supervision, Software, Resources, Project administration, Methodology, Investigation, Funding acquisition, Formal analysis, Data curation, Conceptualization.

## Declaration of competing interest

The authors declare that they have no known competing financial interests or personal relationships that could have appeared to influence the work reported in this paper.
